# Automatic Background Knowledge Selection for Matching Biomedical Ontologies

**DOI:** 10.1371/journal.pone.0111226

**Published:** 2014-11-07

**Authors:** Daniel Faria, Catia Pesquita, Emanuel Santos, Isabel F. Cruz, Francisco M. Couto

**Affiliations:** 1 LASIGE, Faculdade de Ciências, Universidade de Lisboa, Lisboa, Portugal; 2 Departamento de Informática, Faculdade de Ciências, Universidade de Lisboa, Lisboa, Portugal; 3 ADVIS Lab, Department of Computer Science, University of Illinois at Chicago, Chicago, United States of America; Nazarbayev University, Kazakhstan

## Abstract

Ontology matching is a growing field of research that is of critical importance for the semantic web initiative. The use of background knowledge for ontology matching is often a key factor for success, particularly in complex and lexically rich domains such as the life sciences. However, in most ontology matching systems, the background knowledge sources are either predefined by the system or have to be provided by the user. In this paper, we present a novel methodology for automatically selecting background knowledge sources for any given ontologies to match. This methodology measures the usefulness of each background knowledge source by assessing the fraction of classes mapped through it over those mapped directly, which we call the mapping gain. We implemented this methodology in the AgreementMakerLight ontology matching framework, and evaluate it using the benchmark biomedical ontology matching tasks from the Ontology Alignment Evaluation Initiative (OAEI) 2013. In each matching problem, our methodology consistently identified the sources of background knowledge that led to the highest improvements over the baseline alignment (i.e., without background knowledge). Furthermore, our proposed mapping gain parameter is strongly correlated with the F-measure of the produced alignments, thus making it a good estimator for ontology matching techniques based on background knowledge.

## Introduction

Ontology matching is a task of critical importance in the context of the semantic web that has applications in fields such as ontology engineering and information integration [1]–[9]. It has gained particular relevance in the life sciences domain due to the prominent role ontologies have taken in representing knowledge in this domain [10]–[11]. The substantial overlap between existing biomedical ontologies [12], makes ontology matching essential for integrating their information and ensuring interoperability between them.

Ontology matching consists of finding mappings (i.e., correspondences) between semantically related entities belonging to different ontologies to produce an alignment (i.e., a set of mappings) between the ontologies [1]. There are various methods for finding these mappings, and they can be classified according to their granularity (entity-level vs. structural-level) or their interpretation of the input data (syntactic, external, or semantic) [1].

External ontology matching methods rely on the use of background knowledge sources to provide additional structural or lexical information that enables new mappings to be derived between the ontologies being matched [3]. Potential sources of background knowledge include other ontologies or thesauri [13]–[19], lexical databases [19]–[21], and even textual corpora [22] and websites [23]. Ontology matching systems explore these sources to find synonyms and spelling variants for the concepts being matched [16], [19]–[20], to support word sense disambiguation [21], to provide translations [24], or to perform indirect matching [13]–[15], [17]–[19].

Indirect matching consists in matching each of the input ontologies to one or more external ontologies, then deriving mappings between concepts of the input ontologies that are mapped to a common concept (or to related concepts) in the external ontology. This strategy was first proposed by [13] to match anatomy ontologies, then extended by [14] to derive semantic relations other than equivalence. More recently [17] formalized the use of several external ontologies and the combination of direct and indirect matching strategies. Combining direct and indirect matching has since become a common and effective ontology matching strategy, particularly in the life sciences domain. Indeed, it is a strategy shared by many of the leading systems in the biomedical tasks of recent editions of the Ontology Alignment Evaluation Initiative (OAEI) [18]–[19], [25].

Selecting adequate sources of background knowledge is a recognized challenge in ontology matching [26]. While selection has been carried out manually in most ontology matching systems [16]–[20], the ability to select background knowledge sources automatically would undoubtedly increase the applicability of those systems. Given a pair of input ontologies *S* and *T* (for source and target), the goal of an automated background knowledge selection algorithm is to select one or more sources of background knowledge *X_i_* that enable the alignment of *S* to *T*.

One approach previously proposed in this context consists of querying the semantic web for a background ontology *X_i_* for each pair of concepts (*C_S_*, *C_T_*), then using that ontology to infer relationships between the concepts [27]. This strategy is interesting but inefficient, as it requires several millions or even billions of online queries for typical biomedical ontology matching problems (one query per pair of concepts). Furthermore, this strategy is likely to lead to a low precision in domains with complex and ambiguous terminology such as the life sciences, as selection is carried out by a search engine and based on popularity, and thus the background ontologies may not share the domain of the input ontologies.

The approach proposed by [28] addresses both of these issues, as it consists on a more efficient single selection step per matching problem, and it ensures that the domains of the background ontologies overlap with those of the input ontologies by computing the similarities *sim*(*S*, *X_i_*) and *sim*(*T*, *X_i_*). However, this approach does not ensure that the background knowledge sources are effective for matching the input ontologies, which would require that *X_i_* overlaps with *S*∩*T*, or more concretely that the background alignments *A*(*S*, *X_i_*) and *A*(*T*, *X_i_*) have common *X_i_* concepts.

The most recent approach to background knowledge selection addresses this drawback by using the effectiveness of the background knowledge sources as the criterion for selection [29]. The authors defined effectiveness as the number of common *X_i_* concepts in the background knowledge alignments *A*(*S*, *X_i_*) and *A*(*T*, *X_i_*), divided by the average size of the ontologies [29]. Thus, this approach ensures not only that the background knowledge source overlaps with *S* and *T* individually, but also that it overlaps with both in a manner that enables their matching. In addition to the effectiveness measure, the authors also present two algorithms for combining multiple background knowledge sources [29]. Of these, the *topKByComplement* algorithm is particularly interesting in that it aims to select the best combination of background knowledge sources, taking into consideration how they complement each other, rather than the best individual sources.

While effectiveness is perfectly suited for measuring the individual contribution of a background knowledge source, most ontology matching systems that use indirect matching strategies combine these with direct strategies. In such a setting, it is necessary to ensure that a background knowledge source *X_i_* contains new knowledge (e.g., synonyms, relationships) beyond that contained in *S* and *T*, and therefore leads to new mappings between them that could not be derived directly. In other words, *X_i_* should not only enable the matching of *S* and *T* (i.e., be effective), but do so in a way that complements their direct alignment (i.e., be useful).

In this paper, we present a novel methodology for automated background knowledge selection that complements the strategies proposed by [28]–[29]. Given a set of background knowledge sources that share the domain of the matching problem, our methodology identifies and selects those that are most useful. Our methodology is based on the concept of mapping gain, which assesses the contribution of a source of background knowledge in an ontology matching problem by computing the fraction of mappings derived with the background knowledge source over those obtained by matching the ontologies directly. We implement this methodology in the AgreementMakerLight (AML) ontology matching framework [9], [19], and evaluate it in seven benchmark biomedical ontology matching tasks from the OAEI competition, testing as background knowledge sources a portion of the Unified Medical Language System (UMLS) [30] and 22 biomedical ontologies selected from the OBO foundry [31]. We will show that the mapping gain of a background knowledge source is directly related to the quality of the results obtained with that source, and that our methodology is both effective and efficient in selecting background knowledge sources.

## Methods

### Mapping Gain

A source of background knowledge is only useful as a mediator for a given matching problem if its use leads to new correct mappings between the ontologies that cannot be found by comparing them directly. While we do not know *a priori* the correctness of the mappings obtained with a given background knowledge source, if the source is reliable, the number of correct mappings should be a high fraction of the total number of mappings. Thus, we can expect the fraction of mappings that were obtained with a background knowledge source over those obtained directly to be correlated to (and thus a suitable estimator of) the usefulness of that source.

Given ontologies *S* and *T*, their direct (baseline) alignment *B*, and their alignment *A* obtained using background knowledge source *X*, we define the mapping gain of *A* given *B* as the number of mappings (M) in *A* that are not in *B*, divided by the number of mappings in *B*:
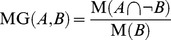
(1)


Note that the mapping gain is deliberately asymmetrical, as it is intended to measure only the contribution of *X*, under the premise that the alignment *A* will be added to *B* to produce a final alignment.

Note also that this definition of mapping gain is only suitable when the true alignment between the source ontology *S* and the target ontology *T* has a cardinality of many-to-many. As we verified empirically, when the cardinality of the alignment is nearly or strictly 1-to-1 (which is common in ontology matching tasks such as those in the OAEI) this definition can lead to an overestimation of the usefulness of *X*. Thus, for such cases, we can define the mapping gain as: 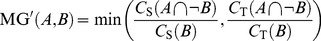
(2)where C_S_ and C_T_ denote respectively the classes in the source and target ontologies. In addition to using the mapping gain to assess the individual usefulness of a background knowledge source, we can use it to assess the combined usefulness of multiple sources. Namely, after selecting the best background knowledge source *X*, we can assess the usefulness of a second source *Y*, by calculating the mapping gain of its alignment *E* given *A* ∪ *B*, where *A* is the alignment obtained with *X* and *B* is the direct alignment. This is precisely the basis of our methodology for automated selection of background knowledge sources.

### Automated Background Knowledge Selection Methodology

Given a set of background knowledge sources, the goal of a selection procedure should be to find the smallest subset of background knowledge sources that leads to the maximum number of mapped classes. However, this problem is formally equivalent to the set cover problem, and thus NP-complete, making it necessary to use a heuristic procedure to produce a solution in useful time [32]. The heuristic we implemented in our methodology is based on the premise that the background knowledge sources with the largest individual mapping gain are more likely to be part of the optimal solution, and furthermore are expected to be more reliable.

Our background knowledge selection methodology consists of two stages: (1) a ranking stage where background knowledge sources are evaluated individually and ranked according to their mapping gain; and (2) a selection stage where background knowledge sources are selected in ranking order, and reevaluated taking into account previously selected sources. The selection stage ensures that additional sources of background knowledge are selected only if they complement the sources previously selected. It makes use of the mapping gain for this end, but follows the same principle as the *topKByComplement* algorithm proposed in [29].

Given two ontologies *S* and *T*, their direct baseline alignment *B*, a set of background knowledge sources *X* =  {*X_1_*,…, *X_N_*} and a minimum mapping gain threshold *k*, the algorithm for our background knowledge selection methodology is the following:

for each X_i_ in X do

A_i_ =  match(S,T,X_i_)

MG'(A_i_,B)

if MG(A_i_,A) <k

remove X_i_ from X

end for

sort X by MG' in descending order

B'  =  B

for each X_i_ in X do

MG'(A_i_,B')

if MG(A_i_,B') <k

remove X_i_ from X

else

B' + =  A_i_

end for

### Implementation

While our automated background knowledge selection methodology can theoretically employ any matching algorithm, there are two important requirements: precision and efficiency. The matching algorithm used must be reasonably precise in order for the mapping gain to be a good estimator of usefulness, and it must be efficient in order for our methodology to be applicable to large sets of background knowledge sources.

We adopted a weighted full-name matching algorithm, the Lexical Matcher, which creates equivalence mappings between classes that have identical labels or synonyms [9], [19]. This algorithm has *O*(*n*) time complexity, and generally leads to a high precision, so it satisfies our requirements. Furthermore, full-name matching is a standard first step in ontology matching, regardless of the overall matching strategy used, so we expect our implementation to have a wide applicability.

We employed the Lexical Matcher both to match ontologies directly (i.e., to create the baseline alignment) and to match them using background knowledge sources. In the latter case, a mapping is considered between two classes of the input ontologies, if both classes have Lexical Matcher mappings to the same class of a background ontology. We assume mapping transitivity because the Lexical Matcher derives only equivalence mappings, though our approach could be extended for other mapping semantics by employing the compose operator [33].

### Similarity Score

One strategy previously proposed for automated selection of background knowledge was based on the similarity between the knowledge source and the ontologies being matched [28]. More concretely given a background knowledge source *X* and two ontologies *S* and *T*, the authors define the following metric for ranking the knowledge source, which we call the *similarity score*:
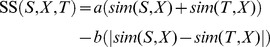
(3)


The authors state that *b* should be slightly lower than *a*, in order to ensure that the background knowledge source is similar to both ontologies rather than to only one of them. Thus, we assume *a* = 1 and *b* = 0.9. Additionally, the authors state that they use the Vector Space information retrieval model to estimate the similarities in equation (3), but provide no other details regarding their implementation. Thus, we adapt the similarity score to the context of our study. We use the Lexical Matcher algorithm [9] to estimate the intersection between each ontology and the background knowledge source, then calculate the similarities using the Jaccard index.

### Effectiveness

Another previously proposed metric for ranking background knowledge was *effectiveness*, which measures the mapping overlap between each of the input ontologies, *S* and *T*, and a background knowledge source *X*, divided by the average size of *S* and *T* [29]. In the context of this study, and given that we're deriving only equivalence mappings, the effectiveness of a background knowledge source *X* can be given by the number of mappings (M) in the alignment *A* obtained with that source, divided by the average size (i.e., number of classes) of the input ontologies:
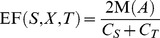
(4)


### Evaluation Tasks

We focus on the biomedical domain to evaluate our methodology due to the critical role that the use of background knowledge plays in ontology matching in this domain. We selected the 7 biomedical ontology matching tasks of the OAEI competition [34] to evaluate our methodology.

The Mouse-Human task from the Anatomy track consists of matching the Mouse Anatomy (MA) ontology to a fragment of the NCI Thesaurus describing the human anatomy. The six tasks from the Large Biomedical Ontologies track consist on matching the FMA ontology to the NCI Thesaurus (FMA-NCI), the FMA ontology to the SNOMED-CT vocabulary (FMA-SNOMED), and the SNOMED-CT vocabulary to the NCI Thesaurus (SNOMED-NCI), in two modalities: a modality where small overlapping fragments of the ontologies are matched, and a modality in which the whole ontologies are matched. The SNOMED-NCI tasks span several life-science domains, whereas the domain of the other five tasks is anatomy.

A reference alignment is publicly available for all seven tasks. However, it is important to note that, while the reference alignment for the Mouse-Human task was manually curated, those for the remaining tasks were automatically derived from the UMLS Metathesaurus [30]. This means that these reference alignments are likely not fully correct or complete, and that there is an inherent bias when using the UMLS Metathesaurus as a background knowledge source in these tasks.

### Background Knowledge Sources

To evaluate our methodology, we collected 22 ontologies from the OBO foundry [31], which covers the domains of the evaluation tasks and are listed in Table 1. In addition to these ontologies, we also used a portion of the UMLS Metathesaurus [30] as a multi-domain biomedical background knowledge source.

**Table 1:** Ontologies used as background knowledge sources.

Ontology Name	Acronym	Domain
Anatomical Entity Ontology	AEO	anatomy
Bilateria Anatomy	BILA	anatomy
Cell Type	CL	anatomy
Chemical Entities of Biological Interest	CHEBI	biochemistry
Common Anatomy Reference Ontology	CARO	anatomy
Foundational Model of Anatomy	FMA	anatomy
Human Disease Ontology	DOID	health
Human Phenotype Ontology	HP	phenotype
Infectious Disease	IDO	health
Mouse Anatomy	MA	anatomy
Minimal Anatomical Terminology	MAT	anatomy
NCI Thesaurus	NCI	health
NIF Cell	NIFC	neuroscience
NIF Dysfunction	NIFD	neuroscience
NIF Gross Anatomy	NIFGA	neuroscience
Ontology for General Medical Science	OGMS	medicine
Phenotypic Quality	PATO	phenotype
Subcellular Anatomy Ontology	SAO	anatomy
Symptom Ontology	SYMP	health
Uber Anatomy Ontology	Uberon	anatomy
Verteberate Homologous Organ Groups	VHOG	anatomy
Vertebrate Skeletal Anatomy Ontology	VSAO	anatomy

In each matching task, we test only background knowledge sources that have a similarity score of at least 0.01%, to ensure that they had a minimal overlap with the ontologies being matched. We exclude the background knowledge sources that corresponded to the ontologies being matched (e.g., Mouse Anatomy and NCI Thesaurus in the Mouse-Human task). We also evaluated the Large Biomedical Ontologies tasks both with and without the UMLS Metathesaurus, so as to account for the bias of using this background knowledge source.

### Evaluation

Given that the reference alignments for the evaluation tasks have near 1-to-1 cardinality, we use a greedy selection algorithm, the Ranked Selector [9], to obtain 1-to-1 alignments prior to evaluating them.

We evaluate the alignments produced by each background knowledge source individually (combined with the baseline alignment) as well as the alignments produced by the combination of background knowledge sources selected by our methodology. The alignments were evaluated in terms of F-measure, which is the harmonic mean of precision and recall, and thus accounts for both type I and type II errors.

We assess the effectiveness of the mapping gain as a parameter for selecting background knowledge sources by measuring the correlation between the mapping gain and the F-measure value. We compute both the mapping gain and the F-measure for each background knowledge source in each ontology matching task and then compute Pearson's correlation coefficient between the two parameters in each task. We also compute the correlation between the similarity score and F-measure, and between the effectiveness parameter and F-measure, to compare these two previously proposed parameters with the mapping gain.

We assessed the performance of our methodology for automatic selection of background knowledge sources by comparing the F-measures of the alignments it produced against the baseline alignments and the optimal alignments (i.e., the alignments obtained with the optimal combination of background knowledge sources) for each task. We studied the effect of the mapping gain threshold on the quality of the results in order to identify a suitable threshold for our methodology.

## Results and Discussion

### Correlation with F-measure

As we detailed in the Methods section, the mapping gain measures the fraction of indirect mappings derived from a background knowledge source over the direct mappings. Thus we know that, for a given baseline alignment *B* and a given background knowledge alignment *A*, the mapping gain multiplied by the precision of the new mappings in *A* gives us the fraction of correct new mappings, which is directly proportional to the recall obtained when combining *A* and *B* (as the recall of *B* is constant):
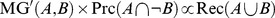
(5)


If we consider that the precision of the new mappings is not significantly different from the precision of *A* and the precision of *B* (which is reasonable considering that the same matching algorithm is employed in both alignments) the relation between mapping gain and F-measure is given by:
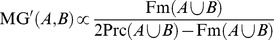
(6)


Thus the mapping gain is related to the F-measure through a non-linear but monotonic function, which means that a higher mapping gain should generally correspond to a higher F-measure if the precision doesn't vary significantly between background knowledge sources. Furthermore, this expression has approximately a linear behavior (with R^2^≥0.99) for F-measure intervals of up to 30%, so we can expect the mapping gain to have a strong linear correlation with the F-measure.

As we can see in Table 2, the mapping gain is indeed strongly correlated with the F-measure for six of the seven evaluation tasks. The one exception is the FMA-NCI whole ontologies task, in which we obtain a low correlation coefficient when including UMLS and a strongly negative correlation coefficient when excluding it. We find that, apart from UMLS, all background knowledge sources have either a neutral or a negative impact on the F-measure (as shown in Table S1), which suggests this result is an artifact. Given that the reference alignment is solely derived from the UMLS Metathesaurus, we can only conclude that it is incomplete, lacking many of the mappings derived from domain ontologies such as Uberon. Indeed, we verified that 17% of the mappings present in the Uberon cross-references (which are manually curated, and thus highly reliable) are absent from the reference alignment, and that this is the reason why Uberon has a negative impact when used as a background knowledge source. This issue is not felt in the corresponding small overlapping fragments task, despite using the same reference alignment, because the fragments are restricted to the regions of the ontologies covered by the reference alignment.

**Table 2:** Correlation between Mapping Gain and F-measure, between Similarity Score and F-measure, and between Effectiveness and F-measure.

Evaluation Task	Correlation with F-measure					
	Mapping Gain		Similarity Score		Effectiveness	
	All	no UMLS	All	no UMLS	All	no UMLS
Mouse-Human	0.998	1.000	0.830	0.884	0.628	0.688
FMA-NCI small	0.997	0.988	0.168	0.716	0.965	0.783
FMA-NCI whole	0.609	−0.985	0.800	−0.938	0.613	−0.840
FMA-SNOMED small	1.000	0.994	0.135	0.925	0.997	0.901
FMA-SNOMED whole	0.996	0.944	0.993	0.789	0.994	0.860
SNOMED-NCI small	0.999	0.987	0.682	0.928	0.994	0.591
SNOMED-NCI whole	0.999	0.972	0.982	0.867	0.993	0.581
Average	0.998	0.981	0.632	0.852	0.929	0.734

Correlation coefficients were computed with all background knowledge sources and with all sources except UMLS. The average was computed excluding the FMA-NCI whole task, as the reference alignment for this task is incomplete, resulting in the negative correlation coefficients observed without UMLS.

If we disregard the FMA-NCI whole ontologies task, the mapping gain has an average correlation coefficient with the F-measure of 0.998 when including UMLS and of 0.981 when excluding it, which clearly shows that the two parameters are strongly correlated. Nevertheless, it is interesting to note that, when excluding UMLS, the correlation coefficients for the whole ontologies tasks are slightly lower than the coefficients for the corresponding small overlapping fragments. This is due to the fact that the small overlapping fragments contain only overlapping regions of the ontologies whereas the whole ontologies are much larger and include non-overlapping regions. Consequently, erroneous matches are more likely in the whole ontologies matching tasks, which means that precision can vary more between background knowledge sources, leading to deviations from the linear behavior and to lower correlation coefficients.

### Comparison with Similarity Score and Effectiveness Parameters

Regarding the effectiveness parameter, we know that the effectiveness of a background knowledge source *X* multiplied by the precision of its alignment *A* is proportional to the recall of *A*:
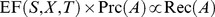
(7)


The key difference to the mapping gain is that the effectiveness doesn't account for the direct alignment *B*. In order to relate effectiveness to F-measure in a typical combined matching strategy, we need to exclude Rec(*A*∩*B*). But whereas the precision of (domain-specific) background knowledge sources can be expected to be reasonably constant, the amount of new knowledge these sources contain for a given matching problem may vary substantially from source to source. A clear illustration of this is observed in the Anatomy task, where the FMA and Uberon have effectiveness scores of the same magnitude (28 and 22% respectively) but the contribution of FMA to the final F-measure is relatively small (1.3%) whereas the contribution of Uberon is high (9.6%). Thus, we can expect the correlation between effectiveness and F-measure to be lower than that of the mapping gain.

As for the similarity score, its relation with the F-measure is even more distant, as it lies on the assumption that, if a background knowledge source *X_1_* is more similar to both input ontologies than a second source *X_2_*, it should enable more mappings between them. While this assumption can generally be expected to be true (at least when the domain overlap of the input ontologies themselves is high), the relation between similarity and generated mappings may or may not be linear, and as we showed for the effectiveness parameter, the relation between all generated mappings and new generated mappings is certainly not linear. Thus, we can also expect the correlation between similarity score and F-measure to be lower than that of the mapping gain.

This is precisely what we observe in Table 2, as the similarity score and effectiveness have lower correlation coefficients with the F-measure in all tasks (except for the aforementioned FMA-NCI whole ontologies task). Furthermore, whereas the mapping gain correctly identified the best background knowledge source in each task (both with and without UMLS), the similarity score and effectiveness failed to do so in several tasks (as shown in Table S1). These results lead to the conclusion that the mapping gain is better suited for identifying useful background knowledge sources than the similarity score or the effectiveness parameter, in a typical setting where direct and indirect matching strategies are combined.

The similarity score has a particularly low average correlation coefficient of 0.632 when using UMLS, although it performs better without UMLS (average coefficient of 0.852). The reason for this is that the similarity score is negatively biased by size differences between the background knowledge source and the input ontologies. Thus, its correlation coefficient is particularly low in the FMA-NCI and FMA-SNOMED small overlapping fragments when using UMLS, because UMLS is substantially larger than the small ontology fragments, and thus has a low similarity score despite being the best background knowledge source (and in fact containing the ontology fragments integrally).

By contrast, the effectiveness has a high average correlation coefficient of 0.929 when using UMLS, and a lower correlation without it (average coefficient of 0.734). The reason for the high correlation with F-measure when using UMLS is because in the 6 tasks where the reference alignment is derived from UMLS, UMLS is unsurprisingly both the best background knowledge and the source that generates more mappings by a substantial margin. When we exclude UMLS, the correlation between effectiveness and F-measure drops significantly by comparison with the mapping gain. This is particularly notable in the case of the SNOMED-NCI tasks, in which several background knowledge sources make small but positive contributions, suggesting that the effectiveness parameter is not very suitable in such a scenario.

### Mapping Gain Threshold

We evaluated our methodology for automated background knowledge selection in six of the seven benchmark biomedical ontology matching tasks, leaving out the whole FMA-NCI matching task due to the incompleteness of its reference alignment. We excluded UMLS from the background knowledge sources in the tasks where the reference alignment was derived from it (i.e., all tasks except Mouse-Human) to avoid introducing that bias in our evaluation.

**Figure 1:**
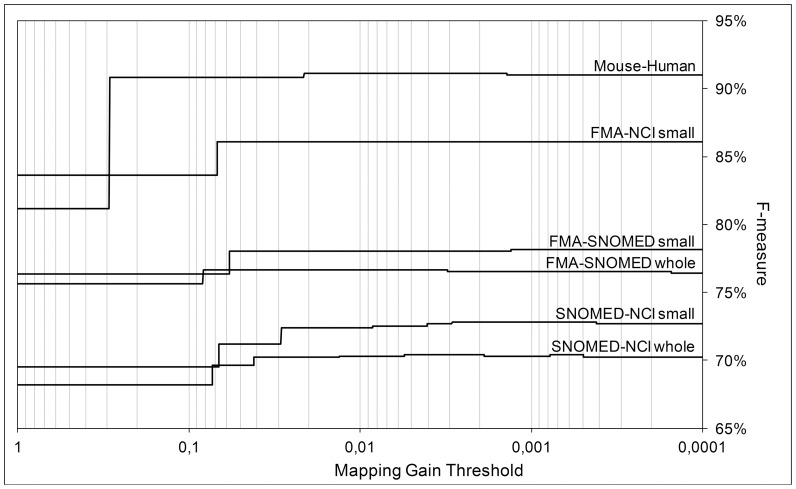
F-measure of the automated background knowledge selection methodology as function of the mapping gain threshold (in descending logarithmic scale) for six ontology matching tasks. The first shift in each line (left-to-right) corresponds to the transition between the baseline alignment and the selection of the best background knowledge source, and subsequent shifts correspond to the selection of additional background knowledge sources. The addition of some background knowledge sources has no visible effect on F-measure.

The performance of our methodology (in terms of F-measure) as a function of the mapping gain threshold is shown in Figure 1. The figure shows that adding multiple background knowledge sources will often lead to better results than adding only the best one, which validates our automated background knowledge selected methodology. The SNOMED-NCI tasks are particularly interesting in that we see substantial contributions from multiple background knowledge sources, due to the fact that these ontologies cover various biomedical domains. However, we also witness that as the mapping gain threshold decreases, the contribution of additional background knowledge sources becomes negative in some tasks, which is tied to the fact that background knowledge sources may also introduce noise [13]. Thus, while the mapping gain varies approximately linearly with the F-measure, it deviates from this behavior at low mapping gain ranges as background knowledge sources become unreliable. This means that there is a practical lower limit for the mapping gain, below which the negative contributions of background knowledge sources outweigh the positive contributions.

In the case of our study, we verified that the lower limit for the mapping gain is 0.3%, and this is the threshold we used in our methodology. However, determining this limit *a priori* for any ontology matching problems is all but impossible, as there is no way of knowing the precision of the new mappings generated by a background knowledge source without using a reference alignment. Thus, in general it will be necessary to employ a more conservative and "safer" threshold.

We found empirically that 2% is a good general-purpose threshold using our matching setting. Moreover, the difference between the results with this threshold and results with the *de facto* optimal threshold are typically small, as lowering the mapping gain threshold brings diminishing returns (as a lower mapping gain implies a smaller contribution). In the case of our study, the difference in F-measure between results with the optimal 0.3% threshold and results with the safer 2% threshold range from 0 to at most 0.3%, which shows that the contributions of background knowledge sources below the 2% threshold are marginal.

For matching settings that differ significantly from ours, it may be necessary to estimate the precision of the matching algorithms employed (or of the overall matching strategy) and adjust the mapping gain threshold accordingly (e.g., by raising it for lower precision algorithms). Additionally, when the background knowledge sources are less reliable (e.g., general purpose rather than domain-specific sources) the mapping gain threshold should also be adjusted.

### Automated Background Knowledge Selection Results

Table 3 shows the performance of our automated background knowledge selection methodology using the 0.3% mapping gain threshold and compares it with the baseline alignment and with optimal manual background knowledge selection (i.e., the combination of background knowledge sources that leads effectively to the best results).

**Table 3:** Run time and F-measure of our automated background knowledge selection methodology, and F-measure of the corresponding baseline and optimal alignments.

Matching Task	Automated Selection		Baseline	Optimal^1^
	Time (s)^2^	F-measure	F-measure	F-measure
Mouse-Human	120	91.1%	81.2%	91.1%
FMA-NCI small	140	86.1%	83.6%	86.1%
FMA-SNOMED small	190	78.1%	76.3%	78.1%
FMA-SNOMED whole	800	76.5%	75.6%	76.6%
SNOMED-NCI small	600	72.7%	69.5%	72.8%
SNOMED-NCI whole	1500	70.4%	68.2%	70.4%

1 Alignment obtained with the manually selected combination of background knowledge sources that leads effectively to the highest F-measure.

2 Experiments were run in a desktop computer with an Intel(R) Core(TM) i5-4570 CPU @ 3.20GHz and 16 GB RAM.

We observe that our methodology leads to substantial improvements over the baseline alignment, and more importantly, leads to results that are identical to the optimal selection in four tasks and results that differ only by 0.1% in the other two tasks. Furthermore, in these two tasks (FMA-SNOMED whole ontologies, and SNOMED-NCI small fragments) our methodology would have matched the optimal selection at a slightly different threshold (0.31% and 0.29% respectively). These results validate the heuristic procedure behind our selection methodology, as they show that it can consistently find optimal or near-optimal solutions.

In terms of performance, the run times of our methodology are within the range of times taken by ontology matching systems in the corresponding OAEI tasks, despite the fact that we are loading around 20 background ontologies per matching task (some of which are quite large). In fact, reading and processing the information in the input and background ontologies represents over 95% of the run time in each task, so the actual run time of the selection methodology is negligible. Thus, our methodology is in practice applicable to any ontology matching framework and should not create a bottleneck in its performance (other than the bottleneck caused by loading the background ontologies, which is transversal to any selection methodology).

## Conclusions

We have shown that the mapping gain is more strongly correlated with the F-measure than the previously proposed similarity score and effectiveness parameters, in a typical matching setting where direct and indirect matching strategies are combined. We have also shown that, assuming approximately constant precision, there is a monotonic relation between mapping gain and F-measure which means that the mapping gain is indeed a suitable estimator of the usefulness of background knowledge sources. These results validate our premise that, although similarity and effectiveness are suitable for identifying domain-specific background knowledge sources, they are less effective than the mapping gain at ranking them and predicting their usefulness.

The automated methodology that we propose for the selection of background knowledge sources uses the mapping gain not only to assess the individual usefulness of each background knowledge source, but also to assess the collective usefulness of combined sources, in a heuristic procedure that aims to select the optimal combination.

We have shown that this heuristic procedure is effective, as it was able to consistently identify optimal combinations of background knowledge sources, with significant improvements over the baseline alignments. From the performance viewpoint, we also show that our methodology is efficient, with low run times considering the number and size of the background ontologies tested (as well as the size of the ontologies being matched).

We based our methodology on a lexical matching algorithm because this is a standard first step in ontology matching, regardless of the overall matching strategy used. Furthermore, this algorithm meets two critical requirements: high precision and *O*(*n*) time complexity (where *n* is the size of the ontologies being matched). Finally, lexical matching is one of the most effective strategies for matching biomedical ontologies, and the main strategy used for indirect background knowledge matching [13], [17]–[19]. Thus, we expect this strategy to be representative of the biomedical domain, and applicable in general to this domain.

We acknowledge that using lexical matching only is limiting as an ontology matching strategy, but would like to note that, despite the focus of our evaluation, both the concept of mapping gain and our automated selection methodology are applicable with theoretically any ontology matching algorithm or combination of algorithms, and to any domain. The only necessary considerations are that the expected precision of the matching algorithms used needs to be taken into account to select a suitable mapping gain threshold, and that using matching algorithms that have *O*(*n^2^*) or higher time complexity will decrease the efficiency of the methodology. However, efficiency should not be a serious issue for real-world ontology matching problems, as ontology matching is a one-shot offline process.

Extending our methodology to account for matches between ontology properties in addition to classes is trivial, and extending it for cases where the desired alignment does not have 1-to-1 cardinality requires only adopting the broader definition of mapping gain we presented in equation (1). It should also be straightforward to implement our methodology with pre-computed mappings, such as those in BioPortal [35]. Finally, the mapping gain can also be used to assess the usefulness of specific direct matching strategies such as synonym-derivation [36].

## Supporting Information

Table S1 F-measure, Mapping Gain, Similarity Score and Effectiveness for each background knowledge source in each ontology matching task.
(XLS)
